# Comparison of CAT and short forms for PROMIS pain and physical health domains in children with sickle cell disease

**DOI:** 10.1186/s41687-023-00553-3

**Published:** 2023-02-14

**Authors:** Sadie F. Mason, Mahua Dasgupta, Kathryn E. Flynn, Pippa M. Simpson, Ashima Singh

**Affiliations:** 1 Department of Pediatrics, Medical College of Wisconsin, Milwaukee, WI USA; 2 Children’s Research Institute, Children’s Wisconsin, Milwaukee, WI USA; 3 Division of Quantitative Health Sciences, Department of Pediatrics, Medical College of Wisconsin, Milwaukee, WI USA; 4 Division of Hematology/Oncology, Department of Medicine, Medical College of Wisconsin, Milwaukee, WI USA

**Keywords:** Sickle cell disease (SCD), Pediatrics, Patient-Reported Outcomes Measurement Information System (PROMIS), Computer adaptive testing (CAT), Short form (SF)

## Abstract

**Background:**

Pain and physical health domains included in Patient-Reported Outcomes Measurement Information System® (PROMIS®) can be administered as short forms (SF) or as computer adaptive tests (CAT). CAT is ideal in many settings but cannot be administered without specialized technology. We compared SF and CAT to identify items for customized SFs to improve the SF performance for children with sickle cell disease (SCD).

**Methods:**

Eligible children 8–17 years old were administered CATs for 5 domains of physical health and 2 domains of pain, followed by any items on the corresponding SF that were not included in the CAT assessments. We describe the range of scores on the CAT and SFs, including the percentage of participants with floor or ceiling effects using the SF. The agreement and correlation between CAT and SF scores were assessed using Bland–Altman plots. Items frequently offered on CAT that had variable responses and were not already present on SF are recommended as additional items for customized SFs.

**Results:**

Among 90 children with SCD, there were strong correlations between CAT and SF scores (Concordance Correlation Coefficient > 0.8) however, the SFs for fatigue, mobility, strength impact, pain behavior, and pain interference had substantial floor/ceiling effects. Fatigue, mobility, physical stress experience, and pain behavior domains had items that were frequently offered on CAT, variable responses, and were not present on the SF.

**Conclusions:**

Adding items to the SFs for the fatigue, mobility, physical stress experience, and pain behavior domains may improve these domains’ SFs performance for children with SCD.

## Background

Sickle cell disease (SCD), a genetically inherited blood disorder, is known to have a significant impact on the quality of life of affected children [1, 2]. This disease affects an estimated 100,000 Americans and occurs in 1 in every 365 Black births in the United States [3]. Anemia and vaso-occlusive pain crises are two of the hallmarks of the disease which directly impact physical health and pain outcomes [4]. The systemic racism that has led to mistrust of the medical community by the Black population is also seen among individuals with SCD. This mistrust, in addition to other barriers including, but not limited to, a disproportionate percentage of this population living below the poverty line and limited access to specialized care, has led to fewer participants in clinical trials, which can make it difficult to evaluate new treatments [5, 6]. Patient reported outcomes (PROs) play a role in learning more about patient experiences. It is therefore important for them to be included and accurately captured in clinical trials evaluating disease management strategies as well as in clinical practice, where standardized questionnaires can foster better communication and tracking of participants’ symptoms and functioning over time [7].

Studies have shown that multiple pain and physical health domains within the Patient-Reported Outcomes Measurement Information System® (PROMIS®) are reliable and valid for children with SCD [8–12]. However, results from our prior work show that some PROMIS short forms (SF) exhibited high floor or ceiling effects for children with SCD. The sample included children with SCD who were not experiencing disease exacerbation. Negatively worded domains such as Pain Behavior (higher score indicating worse health; 8-item SF used) had considerable floor effects (24% at floor, 1.7% at ceiling) [9]. Likewise, positively worded domains such as Physical Strength Impact (higher score indicating better health; 8-item SF used) had considerable ceiling effects (20% at ceiling, 0% at floor) [9]. While floor/ceiling effects at the end of the scale representing worse health may be more concerning, in the context of a life-long chronic disease like SCD, differences between (or changes from) mild to no symptoms are also meaningful and important to be able to distinguish.

Prior work has also examined responsiveness of specific PROMIS domains to changes in a patient’s health status. When looking at the pain domains and the physical stress experience domain, improvement in scores was seen just 7–10 days after an Emergency Department (ED) visit for acute pain [13]. Meanwhile, other domains, including physical activity, physical strength impact, mobility, and fatigue did not show significant improvement until 1–3 months after an ED visit [13]. These findings reflect that there is an opportunity for improvement in the ability of the PROMIS pain and physical health SFs to detect changes in function and quality of life for children with SCD across different times in their disease treatment and evaluation, possibly by creating customized short forms to make the item sets more relevant to this patient population.

While SF assessments have been more commonly used, there are computer adaptive testing (CAT) versions available as well [14, 15]. CAT can better target items at the low and high ends of a domain, to reduce floor and ceiling effects without substantially increasing the total number of items administered [16, 17]. However, SFs offer several other advantages. For one, SFs are more easily given across a variety of locations when the technology might not be available for CAT. Also, there may be benefit in administering the same items to all participants to support longitudinal assessment and to standardize which items are offered [18]. Offering the same items via SF, rather than allowing the CAT algorithm to determine which items are offered, leading to a different set of items each time, may make data more easily interpretable when trying to capture changes in an individual participant’s answers to individual items over time, particularly in the clinical practice setting. A prior study demonstrated PROMIS pediatric measures scores based on CAT and SF were highly correlated [17]. However, this has not been investigated for the pediatric SCD population, as other studies recruited from general pediatric clinics and subspecialty clinics. The objective of our study was to compare the administered PROMIS SF (4-item SF for the fatigue, mobility, and pain interference domains and 8-item SF for the physical activity, physical stress experience, strength impact, and pain behavior domains) and CAT in terms of the total scores and items administered for children with SCD. We also assessed the overlap of CAT administered items and the items included in the longer versions of SF. We hypothesized that there would be high concordance of scores and overlap for the pain domains and for physical stress experience between CAT and administered versions SF administered among children with SCD presenting to a clinic. We also hypothesized that we would identify additional items that can be added to the SFs of specific domains (physical activity, mobility, and fatigue) to improve their performance for children with SCD.

## Methods

### Study population

Our study included a sample of children with SCD, ages 8 to 17 years, in their baseline state of health recruited from the Hematology SCD clinic at a large tertiary care center during the time period from March 2016 to May 2018. Children were considered eligible for the study if they were able to read and speak English and did not have cognitive impairments which could hamper survey comprehension. Consent/assent to participate was obtained at the time of enrollment in the study.

### Data collection

Demographic and clinical characteristics were collected. A parent/guardian of each participant reported Age, Ethnicity, Race, and whether they had been on hydroxyurea therapy for > 1 month. Participants’ specific SCD phenotype were obtained from the clinical chart. Our study included the PROMIS domains related to Pain and Physical Health. There were 5 physical health domains and 2 pain domains included in the study. All PROMIS domains are publicly available and can be accessed online at HealthMeasures.net [19].

#### Physical health domains

PROMIS v1.1 Pediatric Profile 25—Fatigue (“Fatigue”) [20]

PROMIS Pediatric Short Form v1.0—Physical Activity 8a (“Physical activity”) [21, 22]

PROMIS Pediatric Short Form v1.0—Physical Stress Experience 8a (“Physical stress”)

PROMIS v1.1 Pediatric Profile 25—Physical Function Mobility (“Mobility”) [23]

PROMIS Pediatric Short Form v1.0—Strength Impact 8a (“Strength impact”) [21, 22]

The 4-item SFs were used for Fatigue and Mobility. The 8-item SFs were used for Physical Activity, Physical Stress Experience, and Strength Impact.

#### Pain domains

PROMIS Pediatric Short Form v1.0—Pain Behavior 8a (“Pain behavior”) [24]

PROMIS v1.1 Pediatric Profile 25—Pain Interference (“Pain interference”) [25]

The 4-item SF was used for Pain Interference while the 8-item SF was used for Pain Behavior.

The participants were first administered CAT. The CAT version required a minimum of 5 items to be answered; the CAT questionnaire ends when the SE is below the threshold of 4 or when there are 12 items (or the maximum number for the domain) that have been offered. Once the CAT stopped, the patient was asked to complete any items on the SF for that domain that had not already been offered with the CAT. If they were administered a SF item as part of the CAT, they were not administered that item again. This method is identical to the one used by Varni et al. [17] for their study comparing the psychometric properties of different scoring and administration options for pediatric PROMIS domains. Study data were collected and managed in English using Research Electronic Data Capture (REDCap) tools hosted at our institution [26, 27]. REDCap (Research Electronic Data Capture) is a secure, web-based software platform designed to support data capture for research studies, providing (1) an intuitive interface for validated data capture; (2) audit trails for tracking data manipulation and export procedures; (3) automated export procedures for seamless data downloads to common statistical packages; and (4) procedures for data integration and interoperability with external sources [26, 27]. Participants completed the CAT or SF via a tablet in clinic, a link sent to their home email address, or via a phone call after the visit.

### Analyses

#### Scores from the CAT and SF

Item Response Theory (IRT) was used for both CAT and SFs. The SFs were scored based on the response patterns and calibration statistics of the IRT models. IRT is a family of mathematical models that calibrates scoring parameters based on how likely people with different levels of the measured trait are to endorse an item conditional on their level of the health trait being assessed [28]. We graphically illustrate the distributions of scores using histograms for each domain and administration method. We report the percentage of participants with floor and ceiling effect using the SF for each domain. Floor and ceiling effects were calculated as the minimum and maximum scores possible on each SF. Additionally, we used Bland–Altman plots to determine the agreement between CAT and SF and the average difference in scores between the two administration modes. Our Bland–Altman plots considered CAT the gold standard, placed along the x-axis and the difference between CAT and SF along the y-axis. The thick red dotted line is their zero line where no difference between the scores is seen. We also report the Concordance Correlation Coefficient (CCC) where a CCC greater than 0.8 suggests strong correlation [29].

#### Precision of CAT and SFs

We determined the proportion of participants with a standard error (SE) less than 4 for all domains. High SEs are reflective of less precision. We also plotted the SE with scores for both CAT and SF to illustrate the distribution of SE with scores.

#### Overlap of items between CAT and SF

We classified items as frequently offered by CAT if they were answered by > 50% of participants. We evaluated how many of the frequently offered items were not present on the administered SFs. We also determined if these items were present on alternate versions of SF already available for some domains including the 8-item SF for the mobility domain and the 10-item SF for the fatigue and pain interference domains. We recommended adding the frequently offered CAT items to SF for children with SCD if the specific items had at least 15% of subjects choosing something other than the lowest or highest response option.

## Results

There were 90 children with SCD who were administered CAT and subsequently completed the SF. Table 1 shows the demographic and clinical characteristics of our study cohort.

**Table 1:** Demographic and clinical characteristics of participants included in our study

Patient characteristics	All [N = 90] n (%)
Age (year), mean (SD) at Completion	13.2 (3.1)
Age, N (%)	
Age—Participants ages 8–12yrs, N (%)	39 (43.3%)
Age—Participants > 12yrs, N (%)	51 (56.7%)
Sex, female, N (%)*	44 (48.9%)
Race, N (%)	
Race, Black, N (%)	89 (98.9%)
Race, Multiracial (Black and White), N (%)	1 (1.1%)
Ethnicity, Hispanic, N (%)**	4 (4.4%)
Taking Hydroxyurea for > 1mo, N (%)^Ϯ^	55 (61.1%)
Genotype, N(%)***	
Hgb SS	50 (55.6%)
Hgb SC	15 (16.7%)
Hgb SBO Thalassemia	1 (1.1%)
Hgb SB + Thalassemia	1 (1.1%)
Other	2 (2.2%)

*Sex missing for 5

**Ethnicity missing for 23

^Ϯ^Hydroxyurea Missing for 5

***Genotype missing for 21

### Physical health domains

#### Fatigue

**Fig. 1:**
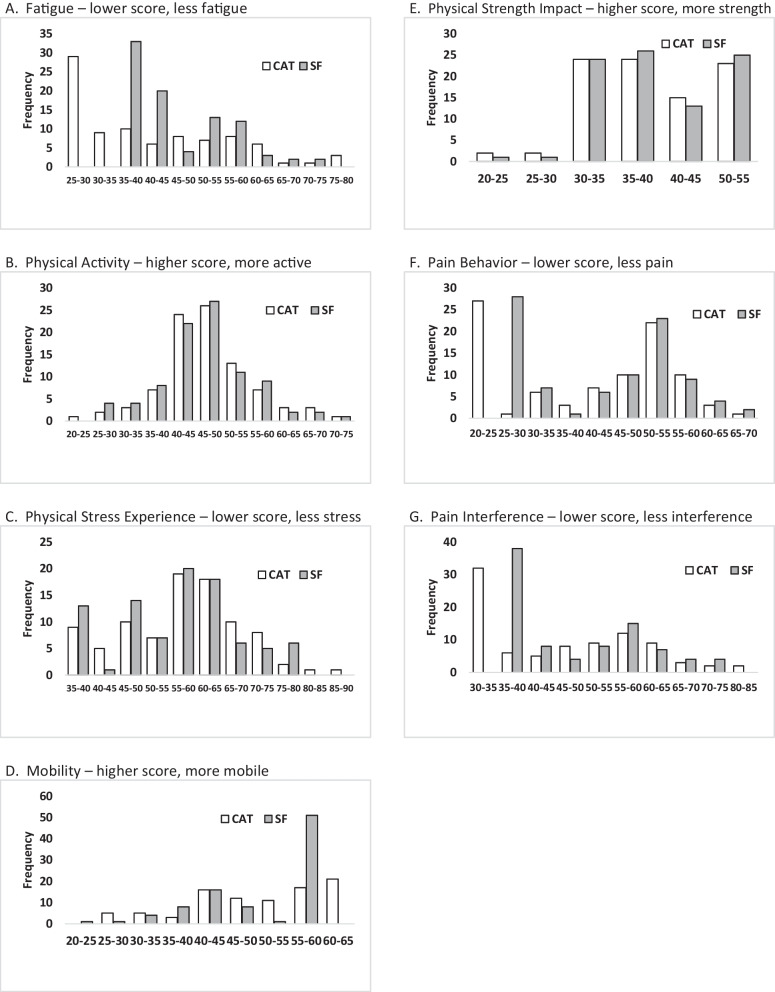
Distribution of scores for CAT and SF for each Domain Included in the Study

Figure 1A shows the distribution of CAT and SF scores for the study participants. Scores for the Fatigue domain ranged from 25.6 to 78.1 for CAT and from 35.4 to 71.9 for SF, a difference in range of over 1 standard deviation (16 points). This domain had a substantial floor effect when using the SF with 37% of participants having the lowest possible score. When using CAT, 45% of the participants had a score lower than the floor score of SF (Table 2).

**Table 2:** CAT and SF Scores, Paired T-test Value, and Floor and Ceiling Effect

Domain	CAT				SF					
	Mean (SD)	Range of scores (Min–Max)	N	N (%) of Pts outside floor-ceiling range**	Mean (SD)	Range of scores (Min–Max)	N	N(%) of Pts at floor	N(%) of Pts at ceiling	Mean (SD) of paired difference CAT-SF
*Physical Health*										
Fatigue	40.97 (14.66)	25.6–78.1	88	40 (45.5)	45.34 (10.3)	35.4–71.9	89	33 (37.1)	0 (0.0)	− 4.38 (6.14)
Physical Activity	47.01 (8.61)	23.5–72.2	90	4 (4.4)	46.49 (8.38)	28.8–71.7	90	4 (4.4)	1 (1.1)	0.53 (1.71)
Physical Stress Experience	57.41 (11.59)	36.1–86.1	90	9 (10.0)	56.52 (10.83)	39.4–79.4	90	13 (14.4)	0 (0.0)	0.89 (4.87)
Mobility	50.04 (10.11)	27.8–61.7	90	29 (32.2)	50.37 (8.65)	24.8–57.1	90	0 (0.0)	51 (56.7)	− 0.33 (4.38)
Physical Strength Impact	40.91 (9.08)	22.6–54.6	90	23 (25.6)	41.1 (8.96)	22.1–54.3	90	1 (1.1)	25 (27.8)	− 0.19 (2.29)
*Pain*										
Pain Behavior	41.56 (13.58)	23.9–69.8	90	28 (31.1)	42.51 (13.41)	26–69.7	90	28 (31.1)	2 (2.2)	− 0.95 (3.78)
Pain Interference	46.31 (13.72)	32.2–83.1	88	35 (39.8)	47.75 (11.68)	36.7–74	90	38 (42.2)	4 (4.4)	− 1.44 (3.42)

^**^We use Floor and Ceiling effect calculated from SF

N is used to calculate the respective percentage

**Fig. 2:**
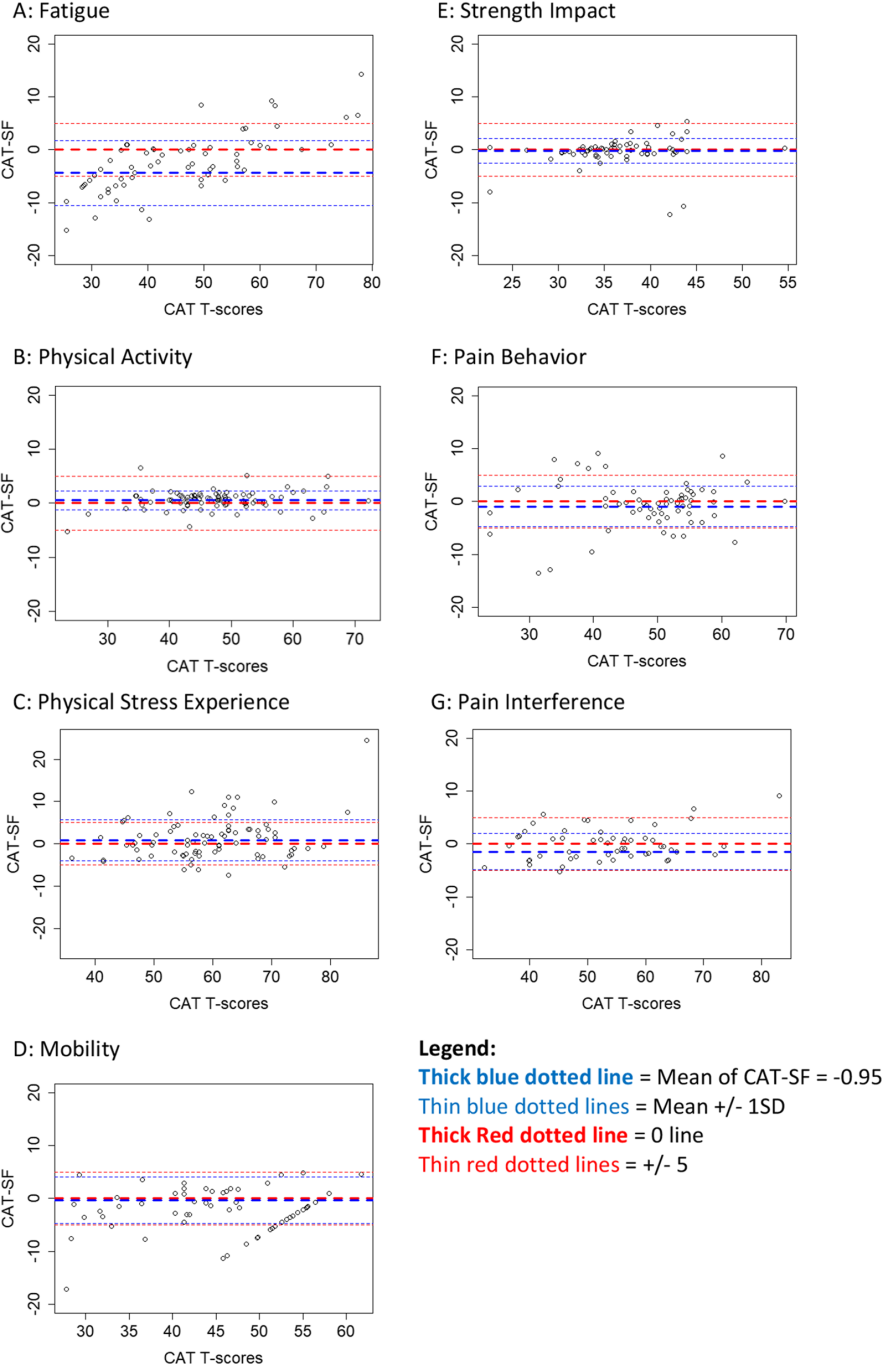
Bland–Altman plots determining agreement between CAT and SF for each domain

The agreement analysis estimated that CAT scores for this domain were 4.38 points lower than the SF scores. The Bland Altman plot (Fig. 2A) also shows that as the CAT scores increased, the direction of this difference changes with CAT scores being higher than the SF scores. The CCC of 0.833 shows strong correlation. While no individual had a SE less than 4 on the SF, 39% of participants had a SE less than 4 on CAT. The mean SE for CAT and SF were 4.43 and 5.71 respectively (Table 3).

**Table 3:** CAT and SF SEs

Domain	CAT		SF	
	Mean SE (overall)	Proportion of Participants with SE < 4 N (%)	Mean SE (overall)	Proportion of Participants with SE < 4 N (%)
*Physical Health*				
Fatigue	4.43	34 (39%)	5.71	0
Physical Activity	2.80	86 (96%)	2.54	85 (94%)
Physical Stress Experience	3.90	64 (71%)	4.33	48 (53%)
Physical Function/Mobility	4.63	40 (44%)	5.67	25 (28%)
Physical Strength Impact	3.48	67 (74%)	3.33	65 (72%)
*Pain*				
Pain Behavior	2.77	63 (70%)	2.85	59 (66%)
Pain Interference	4.26	45 (51%)	5.00	26 (30%)

Participants who completed the CAT answered 5–12 items with an average of 10 items (SD = 3) on CAT for this domain. Of note, there were two participants who did not complete the CAT for this domain but did complete CAT for other domains. Of the frequently offered items on CAT (those that were answered by > 50% of our cohort), 2 were on the 4-item SF and 7 were on the 10-item SF. There were 2 additional items answered by at least 50% of our cohort that had variable responses and were not present on either version of the SF (Table 4a).

**Table 4:** Items to Add to the (a) 10 item SF—Fatigue, (b) 8 item SF—Physical Stress Experience, (c) 8 item SF—Mobility, (d) 8 item SF—Pain Behavior

Item	% of Participants who answered on CAT
*(a)*	
In the past 7 days I needed to sleep during the day	60.23
In the past 7 days I felt tired	56.82
*(b)*	
In the past 7 days, My breathing was fast	61.11
In the past 7 days, My breathing was fast, even when I was not exercising or playing hard	58.89
In the past 7 days, I felt dizzy	50
*(c)*	
In the past 7 days I could run a mile	60
*(d)*	
In the past 7 days, when I was in pain I took breaks from what I was doing	92.22

© 2008–2022 PROMIS Health Organization. Reprinted with permission

#### Physical activity

Figure 1B shows the distribution of CAT and SF scores for the study participants. Scores for the Physical Activity domain are found in Table 2 and ranged from 23.5 to 72.2 for CAT and from 28.8 to 71.7 for SF. This domain did not have significant floor or ceiling effects for the SF, with just 4% of participants at the floor and 1% at the ceiling. When using the CAT, only 4% of participants had scores outside the floor or ceiling of the SF (Table 2).

The agreement analysis estimated that CAT scores in this domain were 0.526 points higher than the SF scores. The Bland–Altman plot (Fig. 2B) reflects this similar distribution, and the CCC for this domain was 0.978 indicating strong correlation between the two measures. On both CAT and SF, the majority (96% on CAT and 94% on SF) of participants had a SE less than 4. The mean SE for CAT and SF were 2.8 and 2.54 respectively (Table 3).

Participants who completed CAT answered 5–10 (maximum number in the domain) items on the CAT for this domain with an average of 5 items answered (SD = 1). Of note, there was one participant who did not complete the CAT for this domain but completed CAT for other domains. In our data collection, we offered the 8-item SF version. There were 5 items that were offered to > 50% of the participants in the CAT, and all were present on the 8-item SF.

#### Physical stress experience

Figure 1C shows the distribution of CAT and SF scores for the study participants. Scores for the Physical Stress domain are found in Table 2 and ranged from 36.1 to 86.1 for CAT and from 39.4 to 79.4 for SF. This domain has some degree of floor effects when using the SF with 14% of participants having the lowest possible score. When using the CAT, 10% of participants had a score lower than the floor score for SF (Table 2).

The agreement analysis estimated that CAT scores in this domain were 0.89 points higher than the SF scores. The Bland–Altman Plot (Fig. 2C) shows this wide distribution of scores and the strong correlation between scores with a CCC of 0.903. There were 71% and 53% of participants who had a SE less than four on the CAT and SF respectively. The mean SE for CAT and SF were 3.9 and 4.33 respectively (Table 3).

Participants answered 5–12 items on the CAT for this domain with an average of 8 items answered (SD = 3). Of the frequently offered items on CAT, 2 were on the 8-item SF. Three other items were offered > 50% of the time on CAT and had variable responses. We recommend adding these items to physical stress experience SF for children with SCD (Table 4b).

#### Mobility

Figure 1D shows the distribution of CAT and SF scores for the study participants. Scores for the Mobility domain are found in Table 2 and ranged from 27.8 to 61.7 for CAT and from 24.8 to 57.1 for SF. This domain had substantial ceiling effect, when using the SF, with 56% of participants having the maximum possible score. When using CAT, there were 32% who had a score higher than the ceiling score of SF (Table 2).

The agreement analysis estimated that CAT scores in this domain were 0.33 points lower than the SF scores. The Bland–Altman plot (Fig. 2D) also shows that there is a linear increase in scores on the CAT at a certain point, reflecting the more dynamic range captured by the CAT. There is strong correlation between scores with a CCC of 0.891. On the SF, 28% of participants had a SE less than while 44% of them had a SE less than 4 on CAT. The mean SE for CAT and SF were 4.63 and 5.67 respectively (Table 3).

Participants answered 5–12 items on the CAT for this domain with an average of 9 items answered (SD = 3). Of the frequently offered items on CAT, 3 were on the 4-item SF and 5 were on the 8-item SF. There was 1 additional item that was answered > 50% of the time on CAT with variable response that was not offered on either SF (Table 4c).

#### Strength impact

Figure 1E shows the distribution of CAT and SF scores for the study participants. Scores for the Strength Impact domain are found in Table 2 and ranged from 22.6 to 54.6 for CAT and from 22.1 to 54.3 for SF. This domain had moderate ceiling effect, when using the SF, with 27% of participants having the maximum possible score. When using CAT, 25% of participants had a score higher than the ceiling score of SF (Table 2).

The agreement analysis estimated that CAT scores in this domain were 0.19 points lower than the SF scores. The Bland–Altman plot (Fig. 2E) further shows the close correlation between scores with a CCC of 0. 968. The majority of participants had a SE less than 4 for both CAT and SF (74% for CAT; 72% for SF). The mean SE for CAT and SF were 3.48 and 3.33 respectively (Table 3).

Participants answered 5–9 items on the CAT for this domain with an average of 8 items answered (SD = 3). There were 6 items that were frequently offered on CAT. All 6 of the frequently offered items on CAT are included on the 8-item SF.

### Pain domains

#### Pain behavior

Figure 1F shows the distribution of CAT and SF scores for the study participants. Scores for the Pain Behavior domain are found in Table 2 and ranged from 23.9 to 69.8 for CAT and from 26 to 69.7 for SF. This domain had moderate floor effect, when using the SF, with 31% of participants having the minimum possible score. When using CAT, 31% of participants had a score lower than the floor score of SF (Table 2).

The agreement analysis estimated that CAT scores in this domain were 0.95 points lower than the SF scores. While there is a floor effect for this domain on both tests, indicating that some participants do not have pain, when pain is present, there is a similar distribution of scores as seen on the Bland–Altman (Fig. 2F). The strong correlation is also seen with a CCC of 0.958. The majority of participants had a SE less than 4 on both CT and SF (70% for CAT and 66% for SF). The mean SE for CAT and SF were 2.78 and 5.73 respectively (Table 3).

Participants answered 5–10 items on the CAT for this domain with an average of 7 items answered (SD = 2) There were a total of 3 items that were offered to more than 50% of our study participants. Of these, 2 are on the 8-item SF. There was only 1 additional item offered with this frequency; it is not present on either version of the SF (Table 4d).

#### Pain interference

Figure 1G shows the distribution of CAT and SF scores for the study participants. Scores for the Pain Interference domain are found in Table 2 and ranged from 32.2 to 83.1 for CAT and from 36.7 to 74 for SF. This domain had significant floor effect, when using the SF, with 42% of participants having the minimum possible score. When using CAT, 39% of participants had a score lower than the floor score of SF.

The agreement analysis estimated that CAT scores in this domain were 1.44 points lower than the SF scores. The Bland–Altman plot (Fig. 2G) reflects the overall similar distribution with a CCC of 0.958 but also show that a floor effect is present with a larger difference noted between scores as the CAT scores get lower. 51% of participants had a SE less than 4 on CAT while just 30% had a SE less than 4 on SF. The mean SE of CAT and SF were 4.26 and 5 respectively.

Participants answered 5–12 items on the CAT for this domain with an average of 8 items answered (SD = 3). 8 items were offered > 50% of the time on the CAT; 4 of them were offered on the 4-item SF, and all 8 are on the 8-item SF.

## Discussion

This study shows that although there is high correlation and agreement between the scores of CAT and SF tests, the SFs for most domains had high floor/ceiling effects for children with SCD. The distribution of scores indicates that CAT provided more nuanced scoring as it was better able to quantify the participants at the floor and ceiling of the domains. The floor, or ceiling, of the SF isn’t necessarily the floor, or ceiling, of the patient’s health, so CAT’s improved differentiation at the extreme ends of the SF’s scoring capabilities becomes particularly important when attempting to determine disease management strategies for children with life-long chronic conditions such as SCD. On average, our participants answered between 5 and 10 items on CAT across the domains. In adding the recommended items to the SF for particular domains, the length of SFs would range from 8 to 11 items, which is comparable to the longer version of most SFs at 8 items and comparable to CATs with current stopping rules (12 items or SE < 4). Alternative CAT stopping rules are now available for adults, and there is a clear need for these rules for the pediatric banks as well, when the SE is not significantly improving with additional items. We recommend using CAT when possible, but when SFs are needed, we recommend using the longest version available and/or adding specific additional items to the available SFs for the fatigue (2 items to the 10-item SF), physical stress experience (3 items to the 8-item SF), mobility (1 item to the-8 item SF), and pain behavior (1 item to the-8 item SF) domains to improve the performance of these domains’ SFs for children with SCD. We recognize that, while using the longer SF or adding additional questions from CAT does provide more precision in the score, it also increases the question burden on the participant. SFs are static and allow for the same set of questions to be administered over time. Hence precise and longer SFs might be preferable in clinical settings where the clinical team or the participant is interested in responses to specific questions. More work is needed in the pediatric SCD population to determine where the balance between increasing number of questions and increasing precision lies for different clinical and research questions. It should also be noted that, when a customized SF is used, it cannot be scored using available look-up tables, so response pattern scoring, such as provided by the Assessment Center Application Programming Interface (API) service available through HealthMeasures [19], is required.

This study is the first to evaluate the overlap between the CAT and SF measures specifically in the SCD population. It is important to evaluate the measures in this population as SCD is a chronic medical condition that is present at birth and can impact all aspects of a child’s life. Pain and fatigue are common complaints in this patient population that might not be as common in the general pediatric population, so these two domains were of special importance in this study. We ultimately show, similar to previous studies that were focused on participants in general pediatric outpatient and subspecialty clinics [17], that there is a strong correlation between the two testing methods for all the domains studied.

By determining the overlap in items between the CAT and SF, we wanted to see if there were items frequently offered on CATs and not offered on SFs that could be added to the SF for that domain. The ultimate goal in determining this overlap is to create a static form that allows for a greater range of scores at the floor or ceiling. A greater range of scores is essential to be able to track improvements or worsening on specific constructs [30]. Hence it is necessary that the tools used are able to capture a range of outcomes. For example, if a drug is being evaluated to improve the pain interference for children with SCD in their baseline state, we need a tool that can detect decrements or improvements. In our study, we observed ceiling effects when using SFs for mobility and physical strength impact (higher scores indicate better outcomes) and floor effects when using SFs for fatigue, pain behavior, and to a lesser extent, pain interference (lower scores indicate better outcomes). This finding could, as previously stated, limit the ability of these specific SFs to capture improvements from baseline state of health for children with SCD, for the floor of the SF is not always the floor of physical health. Therefore, the use of additional items—or, in the case of pain interference, the longer SF already available—is recommended for these domains when CAT is not feasible or there is a clinical/research-based need to ask the same set of items across and within study participants.

We hypothesized that that there would be a high degree of overlap for items offered in the pain domains (Pain Behavior and Pain Interference) and Physical Stress Experience and less overlap amongst the remaining physical health domains, particularly physical activity, because the CAT item bank has fewer items available. We found, however, that in all domains except for fatigue and pain behavior, the majority of the items offered on the CAT > 50% of the time were also present on the longest available version of the SF. This finding drives the high correlation in scores between the two modes of administration. Both pain domains along with fatigue, mobility, and physical strength impact have high numbers of participants whose scores on CAT fall outside the range of participants at floor and ceiling on SF (Table 2). This result suggests that the CAT is using items that better quantify patient experiences on either end of the spectrum. We would recommend adding the additional items that were frequently offered on CAT to the SFs for these domains (or using the longer version of the SF in the case of physical strength impact and pain behavior) to create an a more precise SF for children with SCD (Table 5).

**Table 5:** SF administered and the number of questions to be added compared to longer SF and number of questions to be added

Domain name	SF administered in study		Longest available SF	
	Number of items on SF	Recommended changes for SCD	Number of items on SF	Recommended changes for SCD
*Physical Health*				
Fatigue	4	Add 8 items	10	Add 2 items
Physical Activity	8	No change	8	No change
Physical Stress Experience	8	Add 3 items	8	Add 3 items
Physical Function/Mobility	4	Add 7 items	8	Add 1 item
Physical Strength Impact	8	No change	8	No change
*Pain*				
Pain Behavior	8	Add 1 item	8	Add 1 item
Pain Interference	4	Add 3 items	8	No change

Our study has some limitations that suggest more areas of investigation in the future. We have a single-site convenience sample, which might limit generalizability; however, it is moderate in size and has a similar distribution to other population-based studies in terms of race, ethnicity, sex, and disease subtype [31].

In conclusion, this study supports the expectation that there is a significant correlation between the PROs scores for CAT, the current gold standard, and SF in individuals with SCD. Particularly for the fatigue, mobility, physical stress experience, and pain behavior domains, adding items to the SF that were frequently offered on CAT might provide an even better static tool for the SCD population. Adding items to those domains’ SF or, when that is not possible, using the already available longer SF version, provides, for this patient population, a more nuanced assessment of quality of life by improving precision and overcoming floor and ceiling effects.

## Data Availability

The datasets used and/or analyzed during the current study are available from the corresponding author on reasonable request.
